# Comparison of mucosal phosphatase activity, phytate degradation, and nutrient digestibility in 3-week-old turkeys and broilers at different dietary levels of phosphorus and phytase

**DOI:** 10.1016/j.psj.2022.102457

**Published:** 2022-12-29

**Authors:** Moritz Novotny, Vera Sommerfeld, Jochen Krieg, Imke Kühn, Korinna Huber, Markus Rodehutscord

**Affiliations:** ⁎ Institute of Animal Science, University of Hohenheim, 70599 Stuttgart, Germany; † AB Vista, 64293 Darmstadt, Germany

**Keywords:** phytate degradation, *myo*-inositol, broiler, turkey, digestibility

## Abstract

A comparison between 3-wk-old female turkeys (B.U.T. 6) and broilers (Ross 308) was performed to study the effects of species, dietary P, Ca, and phytase levels on gut mucosal phosphatase activity, *myo*-inositol hexakisphosphate (InsP_6_) degradation along the digestive tract, digestibility of P, Ca, and amino acids, and concentrations of *myo*-inositol in the digesta and blood. The experimental diets were corn-soybean meal-based and identical for both species. Two dietary P and Ca concentrations (CaP–: 4.1 g P/kg, 5.5 g Ca/kg and CaP+: 9.0 g P/kg, 12.0 g Ca/kg) and 2 levels of phytase supplementation (0 and 1,500 FTU/kg) were used in a 2 × 2 factorial design and fed to the animals for 7 d in their third week of age. Each diet was randomly assigned to 6 broiler and 6 turkey pens, with 10 birds each. After slaughter, blood, digesta from the crop, gizzard, duodenum, lower ileum, and mucosa from the jejunum were collected. When fed CaP– without phytase supplementation, there were no differences between species in gut mucosal phosphatase activity, prececal InsP_6_ disappearance, and P and Ca digestibility, indicating a similar intrinsic capacity for phytate degradation in both species. When fed CaP+ without phytase supplementation, turkeys showed higher prececal InsP_6_ disappearance than broilers. Phytase supplementation increased prececal InsP_6_ disappearance and digestibility of P and Ca in both species. However, the phytase-induced increase in prececal InsP_6_ disappearance was more pronounced in broilers than in turkeys, possibly due to more adequate conditions for phytase activity in the broiler crop. In broilers, phytase supplementation increased amino acid digestibility overall, whereas, in turkeys, it increased with CaP+ and decreased with CaP–. In addition, the relationship between *myo*-inositol concentration in the ileum and blood differed between species, indicating differences in *myo*-inositol metabolism. It was concluded that 3-week-old turkeys and broilers differ in nutrient digestibility and InsP degradation in some segments of the digestive tract but have similar endogenous InsP_6_ degradation when fed low P and Ca diets.

## INTRODUCTION

Most P in plant seeds is bound in the form of phytate (any salt of *myo*-inositol hexakisphosphate (**InsP_6_**)). Phytases and other phosphatases must hydrolyze InsP_6_ before InsP_6_-P can be absorbed in the digestive tract of animals. In non-ruminants, endogenous phytase activity is considered to be inadequate, leading to limited InsP_6_ degradation in the digestive tract. However, the potential of broilers to degrade InsP_6_ by the end of the ileum is high (64–76% prececal InsP_6_ disappearance) (Tamin et al., 2004; Zeller et al., 2015a; Ingelmann et al., 2019), with substantial contributions from endogenous gut mucosal phytase and phosphatases (Sommerfeld et al., 2019). However, supplementation with mineral P sources reduced the potential to degrade InsP_6_ (Shastak et al., 2014; Zeller et al., 2015b; Sommerfeld et al., 2018; Sommerfeld et al., 2019), and the inclusion of mineral P in poultry feed remains an industry standard. This leads to the excretion of unused phytate P, which can cause eutrophication of water bodies (Sharpley, 1999). Mineral P supplements are produced from mined phosphate rock, which is a finite resource. To reduce or avoid the inclusion of unsustainable and expensive mineral P and to reduce P excretion, a common strategy is to supplement poultry feed with exogenous phytases. These enzymes release P from InsP_6_ and may also increase the digestibility of amino acids (**AA**), and bioavailability of cations such as Zn, Fe, Mn, Ca, and Mg chelated with phytate (Rodehutscord et al., 2022).

Several studies have been conducted to better understand the process of InsP_6_ degradation in broiler chickens. However, such studies are scarce for turkeys. In comparative studies between turkeys and broilers, prececal InsP_6_ disappearance was much lower in turkeys than broilers (5.5–15.3% in turkeys vs. 63.8–76.3% in broilers) when exogenous phytase was not supplemented (Ingelmann et al., 2019). Correspondingly, upon exogenous phytase supplementation, prececal InsP_6_ disappearance increased more in turkeys than in broilers, although it remained lower in turkeys (Ingelmann et al., 2019; Olukosi et al., 2020). Other studies have also reported that turkeys were more responsive to phytase supplementation than broilers (Pirgozliev et al., 2007). This finding suggests the existence of fundamental differences between broiler chickens and turkeys. However, species-specific feeds were used in all studies published on this subject, which differed markedly in ingredient composition and nutrient concentrations, such as total P, InsP_6_-P, Ca, and crude protein. Because of the interactions between InsP_6_ degradation and dietary Ca and P concentrations, such differences in diet composition may confound possible species effects. In a study that used the same diet without mineral P supplementation in broiler chickens and turkeys, total tract P retention was 58% in broilers and 39% in turkeys at 4 wk of age (Rodehutscord and Dieckmann, 2005). However, InsP_6_ degradation was not investigated in this study.

The main objective of this study was to compare prececal InsP_6_ disappearance in broilers and turkeys, including the effects of dietary Ca, P, and phytase concentrations, using the same feed for both species. It was hypothesized that broilers would have a higher capability to degrade InsP_6_ than turkeys. Another objective was to determine the traits (gut length, pH of digestive tract contents, and jejunal mucosal phosphatase activity) that might explain potential species differences in their abilities to degrade InsP_6_ and utilize InsP_6_-P.

## MATERIALS AND METHODS

### Birds and Housing

The trial was conducted at the Experimental Station “Unterer Lindenhof” of the University of Hohenheim, Germany. This study was approved in accordance with the German Animal Welfare Legislation by Regierungspräsidium Tübingen, Germany (Project No. HOH 59/19 TE).

A total of 530 female Ross 308 broiler hatchlings and 530 female B.U.T. 6 turkey hatchlings were obtained from commercial hatcheries (Brüterei Süd ZN der BWE-Brüterei Weser-Ems GmbH & Co. KG, Regenstauf, Germany and Gebrüder Böcker Putenbrüterei GmbH, Wallhausen, Germany) and placed in floor pens on wood shavings (broilers in 2 pens, turkeys in 4 pens). During the 14 d post-hatch period, broilers were fed a pelleted diet that met or exceeded the supply recommendations of the Gesellschaft für Ernährungsphysiologie (1999) and contained 240 g CP/kg, 8 g P/kg, 9.6 g Ca/kg, and 12.6 MJ ME/kg, with no phytase added. Turkeys were fed a commercial granulated starter diet (Bio-PSG N10 + Ei + C, Kaisermühle Gänheim Otmar Kaiser GmbH, Arnstein-Gänheim, Germany) containing 305 g CP/kg, 10 g P/kg, 14 g Ca/kg, and 11.4 MJ ME/kg until d 8. From d 8 to 14, turkeys were fed a pelleted starter diet formulated to meet or exceed the feeding recommendations of the Gesellschaft für Ernährungsphysiologie (2004), containing 266 g CP/kg, 9 g P/kg, 14.5 g Ca/kg, and 12.3 MJ ME/kg without added phytase. On d 15, 240 birds of both species were allocated in groups of 10 to 48 perforated floor pens (1.15 m × 2.3 m for broilers and 3 m × 4 m for turkeys). The other animals remained in the pens on wood shavings for an additional 21 d, after which they underwent a similar trial reported in a companion paper (Novotny et al., 2023). Each pen was equipped with 2 feeding troughs and 1 drinker. All animals were weighed on a pen basis, and similar body weights per pen for all treatments were maintained within each species. Each pen was allocated in a randomized complete block design to 1 of 4 experimental diets, with 6 pens per diet and species, and feed and water were provided for ad libitum consumption until slaughter on d 21. The light program was 24L:0D for the first 3 d and 18L:6D from d 4 until the end of the experiment. The temperature was 34°C for the first 3 d and then gradually lowered to 26°C on d 21. The animals were inspected twice daily for abnormal behavior and overall health. No mortality was observed during the experimental period.

### Experimental Diets and Treatments

The experiment was designed as a 2 × 2 × 2 factorial arrangement of treatments (2 species, 2 Ca and P levels (**CaP**), and 2 levels of phytase (**PHY**) addition). In the experimental phase, both species were fed the same experimental diets (Table 1) and were concurrently housed in the same barn in order to not confound the species comparison by differences in diet composition or environmental conditions. The experimental diets were based on corn and soybean meal and formulated to meet the supply recommendations for turkeys (Gesellschaft für Ernährungsphysiologie, 2004), except for P and Ca. The CaP+ diets were supplemented with monocalcium phosphate (**MCP**) and limestone to achieve P and Ca concentrations of 9.0 g/kg and 12.0 g/kg, respectively. No mineral phosphate was added to the CaP– diets, resulting in calculated P and Ca concentrations of 4.1 g/kg and 5.5 g/kg, respectively. Sand was used at the expense of MCP and limestone. In the PHY+ diets, a modified E. coli-derived 6-phytase (Quantum Blue, AB Vista, Marlborough, United Kingdom) was added at 1,500 FTU/kg. PHY– diets did not contain added phytase. All the diets contained 5 g/kg TiO_2_ as an indigestible marker. To achieve uniformity of all diets, ingredients except MCP, limestone, sand, and PHY were mixed in one lot. This basal lot was divided into 2 parts. One part was supplemented with MCP and limestone, and the other was supplemented with sand. Each of these mixes was split into 2 parts, and one was supplemented on top with the enzyme, while the other remained without it. The diets were pelleted without steam through a 3-mm die. Mixing and pelleting were performed at the certified feed mill of the Agricultural Experimental Station of the University of Hohenheim. The formulated concentrations of P, InsP_6_-P, Ca, and phytase activity were confirmed by the analyses (Table 2). The phytase activity in the PHY+ diets was lower than the calculated value but similar in both the phytase-containing diets (approximately 1,100 FTU/kg).

**Table 1:** Ingredient composition and calculated nutrient concentrations of the experimental diets.

Ingredient, g/kg	CaP–PHY–	CaP–PHY+	CaP+PHY–	CaP+PHY+
Corn	436.8	436.8	436.8	436.8
Soybean meal	416.5	416.5	416.5	416.5
Rapeseed meal	40.1	40.1	40.1	40.1
Soybean oil	43.2	43.2	43.2	43.2
L-lysine-sulfate	7.5	7.5	7.5	7.5
DL-methionine	3.8	3.8	3.8	3.8
L-threonine	0.7	0.7	0.7	0.7
L-valine	0.3	0.3	0.3	0.3
Choline chloride	2.0	2.0	2.0	2.0
NaCl	1.0	1.0	1.0	1.0
NaHCO₃	4.6	4.6	4.6	4.6
Vitamin mix^1^	2.0	2.0	2.0	2.0
Mineral mix^2^	0.5	0.5	0.5	0.5
Titanium dioxide	5.0	5.0	5.0	5.0
Limestone	7.3	7.3	12.9	12.9
Monocalcium phosphate	0.0	0.0	23.1	23.1
Sand	28.7	28.7	0.0	0.0
Calculated (g/kg):				
P	4.1	4.1	9.0	9.0
Ca	5.5	5.5	12.0	12.0
Crude protein	253	253	253	253
Phytase (FTU/kg)	0	1,500	0	1,500

1 Vitamin mix (MIAVIT GmbH, Essen (Oldb.), Germany), provided per kg of complete diet: 10,000 IU vitamin A, 3,000 IU vitamin D3, 30 mg DL-α-Tocopherylacetate, 2.4 mg vitamin K3, 3 mg vitamin B1, 6 mg vitamin B2, 6 mg vitamin B6, 30 μg vitamin B12, 50 mg nicotinic acid, 14 mg pantothenic acid, 1 mg folic acid, 0.1 mg biotin.

2 Mineral mix (Gelamin, Gesellschaft für Tierernährung mbH, Memmingen, Germany), provided per kg of complete diet: 50 mg calcium from calcium carbonate, 80 mg manganese from manganese-(II)-oxide, 60 mg zinc from zinc-oxide, 25 mg iron from ferrous-(II)-carbonate, 7.5 mg copper from cupric-(II)-sulphate pentahydrate, 0.6 mg iodine from calcium iodate, 0.2 mg selenium from sodium selenite.

**Table 2:** Analyzed composition of the experimental diets.

	Treatments^1^			
Analyzed composition (g/kg^2^)	CaP– PHY–	CaP– PHY+	CaP+ PHY–	CaP+ PHY+
InsP_6_ (mmol/kg as fed)	12.7	12.9	12.4	12.4
InsP_5_ (mmol/kg as fed)	1.6	1.7	1.6	1.6
*Myo*-inositol (µmol/g)	1.8	1.8	1.8	1.8
InsP_6_-P	2.5	2.4	2.4	2.4
P	4.5	4.5	9.7	9.9
Ca	5.7	5.7	12.2	12.3
Crude protein	264	264	257	264
Arg	17.5	17.9	17.7	17.8
His	7.3	7.4	7.4	7.5
Ile	11.6	12.0	11.8	11.8
Leu	22.1	22.5	22.4	22.5
Lys	18.9	19.5	19.2	19.4
Met^3^	7.7	7.9	7.9	7.9
Phe	13.1	13.3	13.3	13.4
Thr	10.8	11.0	11.0	11.1
Val	12.9	13.2	13.0	13.1
Phytase (FTU/kg)	< 50	1130	< 50	1080

1 Calculated composition: CaP–, 4.1 g P/kg and 5.5 g Ca/kg; CaP+, 9.0 g P/kg and 12.0 g Ca/kg; PHY–, no supplemented phytase; PHY+: 1,500 FTU/kg supplemented phytase.

2 Unless stated otherwise.

3 Methionine determined as methionine sulfone.

### Procedures and Sampling

Animals and feed were weighed on d 14 and 21 on a pen basis, and ADG, ADFI, and gain per feed (**G:F**) were calculated. On d 21, the feed troughs of the pens were removed 2 h before slaughter according to a fixed time schedule and returned 1 h before slaughtering to standardize the gut fill. The birds were stunned using a gas mixture consisting of 35% N_2_, 35% CO_2_, and 30% O_2_. Two stunned birds per pen were randomly selected and weighed. They were killed by decapitation and trunk blood was collected in tubes containing sodium fluoride. Blood samples were centrifuged for 10 min at 2,500 ×*g* to obtain plasma. One of the birds was eviscerated, the small intestine and ceca were spread out on a 1 cm grid, and a picture was taken to determine the length of the intestine sections later. The jejunum was dissected from the second bird, opened longitudinally, and flushed with phosphate-buffered saline. Mucosa samples were stripped off with microscopic slides, shock-frozen in liquid nitrogen, transported on dry ice to the laboratory, and stored at −80°C until further analysis. The remaining 8 stunned birds from each pen were asphyxiated using CO_2_. From all 10 birds in a pen, crop, gizzard, duodenum, and lower ileum, defined as the last two-thirds of the section between Meckel's diverticulum and 2 cm prior to the ileo-ceco-colonic junction, and ceca were excised. Digesta of the crop and gizzard were carefully obtained with a spatula, digesta of the duodenum was gently squeezed out, and the digesta of the ileum and ceca were flushed out using ice-cold double-distilled water. The samples of the respective sections were pooled on a pen basis and frozen at −20°C following the determination of pH values in the contents of the crop, gizzard, and duodenum (InLab Solids, Mettler-Toledo GmbH, Vienna, Austria).

### Sample Preparation and Chemical Analyses

Digesta samples were freeze-dried, pulverized (PULVERISETTE 9; Fritsch GmbH, Idar-Oberstein, Germany), and stored in sealed containers at room temperature. Pulverized feed and digesta samples were analyzed for P, Ca, and Ti using inductively coupled plasma-optical emission spectrometry after wet digestion (Zeller et al., 2015a). Extraction and measurement of InsP_3-6_ isomers were carried out using the method of Zeller et al. (2015a) with modifications described by Sommerfeld et al. (2018) and using high-performance ion chromatography (ICS-3000 system, Dionex, Idstein, Germany). Using this methodology makes separating enantiomers impossible; therefore, we could not distinguish between the D- and L-forms. Furthermore, discrimination of isomers Ins(1,2,6)P_3_, Ins(1,4,5)P_3_, and Ins(2,4,5)P_3_ was not possible because of coelution; therefore, we used the term InsP_3x_ for these InsP_3_ isomers of unknown proportions. *Myo*-inositol (**MI**) in the feed, digesta, and plasma samples was analyzed according to Sommerfeld et al. (2018) using a gas chromatograph/mass spectrometer (Agilent 5977A, Waldbronn, Germany) following a two-step derivatization of the samples. Phytase activity in the feed was analyzed by AB Vista Lab Service (Ystrad Mynach, Wales, UK) using a validated product-specific ELISA method. The results were calculated from a calibration curve with a known activity as determined by the Quantum Blue product analysis, and the activity was expressed as FTU/kg feed. Amino acids were analyzed according to Rodehutscord et al. (2004) using an L-8900 amino acid analyzer (VWR, Hitachi Ltd, Tokyo, Japan) following sample oxidation and acid hydrolysis.

### Mucosal Phosphatase Activity Measurement

For mucosal phosphatase activity measurement, the brush-border membrane (**BBM**) was enriched in the mucosa samples, according to Huber et al. (2015). In brief, mucosal samples of the jejunum were ground using a mortar and pestle under liquid nitrogen and mixed with 4-(2-hydroxyethyl)piperazine-1-ethanesulfonic acid/mannitol buffer (HEPES 2 mmol, mannitol 50 mmol, PMSF 25 mmol). Using a glass potter and homogenizer (homogen^plus^, shuett-biotec GmbH, Göttingen, Germany), samples were homogenized, and enterocytes in the mucosa sample were sheared off at the tight junctions, separating BBM from the basolateral membrane. After this step, the mucosal homogenates were mixed with 1 M MgCl_2_ to precipitate the basolateral membranes, which were subsequently removed by centrifugation. Precipitates containing enriched BBM after the final high-speed centrifugation were resuspended in HEPES/mannitol buffer with protease inhibitors. Aliquots (50 μL) of BBM homogenates were frozen and stored at −80°C until analysis. Protein concentrations of the BBM preparations were determined in triplicate using the Bradford assay (Bradford Reagent, 5 ×, SERVA, Heidelberg, Germany). The activity of phosphatases, including phytases, associated with BBM was measured as described by Gonzales-Uarquin et al. (2020) with modifications. Briefly, BBM preparation (equivalent to 160 µg protein) was added to a mixture of double-distilled water containing 25 µg of sodium phytate (Sirius Fine Chemicals SiChem GmbH, Bremen, Germany) and buffer (pH 5.5 buffer from a test kit (K-PHYT 05/17 assay; Megazyme International, Ireland). After 15 min of incubation at 40°C, the reaction was stopped by adding trichloric acid. A second aliquot of the BBM preparation from the same animal was incubated in the same way, but the stop reagent trichloric acid was added beforehand, thus creating a blind value. Following incubation, free phosphate (**P_i_**) was determined photometrically (655 nm, 40°C, Infinite 200 PRO M NANO+, Tecan Trading AG, Switzerland) using the method described in the K-PHYT test kit. The released P_i_ was calculated by subtracting the respective blind values from the original measurement. The activity of BBM-associated phosphatase is the amount of P_i_ released per gram of BBM protein per minute incubation time at pH 5.5. If insufficient BBM preparation was available for the determination of a blind value, the mean of all blind values of the respective treatment was used to calculate the released P_i_.

### Calculations and Statistical Analysis

Prececal digestibility of P, Ca, AA, and InsP_6_ disappearance was calculated using the marker method and the following equation:

$$y\left(X\right)=100-100\times (X_{\text{digesta}}\times \text{Ti}O_{2\text{feed}})/(\text{Ti}O_{2\text{digesta}}\times X_{\text{feed}})$$

where y is the disappearance or digestibility of X in %; X is the concentration of InsP_6_, P, Ca, or AA in the feed and digesta; and TiO_2_ is the concentration of TiO_2_ in the feed and digesta.

The data were analyzed with a 3-way ANOVA using the MIXED procedure of the software package SAS (version 9.4; SAS Institute Inc., Cary, NC). Non-normally distributed data were log-transformed. The results are presented as LSmeans and pooled SEM of the untransformed data. The pen was considered the experimental unit. The following model was used.

$$y_{\text{ijkl}}=\mu +\alpha_{i}+\beta_{j}+\gamma_{k}+{\left(\alpha \beta \right)}_{\text{ij}}+{\left(\alpha \gamma \right)}_{\text{ik}}+{\left(\beta \gamma \right)}_{\text{jk}}+{\left(\alpha \beta \gamma \right)}_{\text{ijk}}+\delta_{l}+\varepsilon_{\text{ijkl}}$$

where y_ijkl_ = response variable, µ = overall mean, α_i_ = effect of species (fixed), β_j_ = effect of CaP (fixed), γ_k_ = effect of PHY (fixed), (αβ)_ij_ = interaction between species and CaP (fixed), (αγ)_ik_ = interaction between species and PHY (fixed), (βγ)_jk_ = interaction between CaP and PHY (fixed), (αβγ)_ijk_ is the 3-way interaction between species, CaP, and PHY (fixed), δ_l_ = effect of block (random), and ε_ijkl_ = residual error. Statistical significance was set at *P* < 0.05.

## RESULTS

Analyses of gastrointestinal pH, mucosal enzyme activity, prececal InsP_6_ disappearance, and P and Ca digestibility are shown in Table 3. The pH of the crop content was higher in turkeys than in broilers. It was lower overall in CaP+ treatments than in CaP– treatments, but this effect was greater in turkeys, resulting in a species × CaP interaction (CaP+ turkey: 6.6, CaP+ broiler: 6.0, turkey: 5.7, CaP– broiler: 5.6; *P* < 0.001). In the gizzard content, species and CaP also significantly affected the pH value, but an interaction did not exist. Gizzard pH was lower in turkeys than in broilers (3.8 vs. 4.0, *P* < 0.001) and higher in CaP+ (3.9) than in CaP– (3.8, *P* = 0.025). The pH value of the duodenum content was higher in broilers (6.3) than in turkeys (6.1, *P* < 0.001) but was not affected by the other factors. The phosphatase activity in the jejunal mucosa was only affected by added phytase and was higher in PHY+ (4.7 µmol P_i_/g/min) than in PHY– (3.6 µmol/g/min) (*P* = 0.043). Prececal InsP_6_ disappearance was affected by a 3-way interaction (*P* = 0.013). InsP_6_ disappearance in broilers did not differ from turkeys at CaP–PHY–, but it was higher in turkeys than in broilers (6.3% vs. −3.9%) at CaP+PHY–. In the treatments with added phytase, broilers had a higher InsP_6_ disappearance than turkeys, and the level was lower overall in CaP+ than in CaP–.

**Table 3:** Effect of Ca and P level (CaP) and phytase supplementation (PHY) on prececal calcium (Ca) and phosphorus (P) digestibility, prececal (pc) InsP_6_ disappearance, jejunal endogenous mucosal phosphatase activity, and pH in the crop, gizzard, and duodenum of broilers and turkeys at 21 d of age.

Species	Treatment^1^		pH crop	pH gizzard	pH duodenum	Mucosal phosphatase activity (µmol P_i_/g BBM protein/min.)	pc InsP_6_ disappearance (%)	pc P digestibility (%)	pc Ca digestibility (%)
Broiler	CaP–	PHY–	5.8	4.0	6.3	3.5	29.3^c^	41.1^e^	45.5
		PHY+	6.1	3.9	6.3	4.8	77.9^a^	70.8^a^	57.1
	CaP+	PHY–	5.6	4.0	6.3	3.6	−3.9^e^	44.8^e^	28.1
		PHY+	5.6	4.1	6.3	4.6	62.5^b^	49.8^d^	27.8
Turkey	CaP–	PHY–	6.6	3.8	6.2	4.5	25.3^c^	44.7^e^	43.2
		PHY+	6.5	3.6	6.1	5.7	58.0^b^	61.4^b^	52.7
	CaP+	PHY–	5.7	3.8	6.1	2.9	6.3^d^	56.2^c^	39.5
		PHY+	5.8	3.8	6.1	3.9	32.4^c^	57.5^bc^	39.2

SEM			0.07	0.08	0.04	0.80	3.35	1.87	2.51

*P*-values									
Species			< 0.001	< 0.001	< 0.001	0.836	< 0.001	0.001	0.003
CaP			< 0.001	0.025	0.232	0.104	< 0.001	0.016	< 0.001
PHY			0.318	0.364	0.803	0.043	< 0.001	< 0.001	< 0.001
Species × CaP			< 0.001	0.936	0.341	0.131	0.673	< 0.001	< 0.001
Species × PHY			0.300	0.761	0.561	0.965	< 0.001	< 0.001	0.673
CaP × PHY			0.376	0.106	0.739	0.837	0.237	< 0.001	< 0.001
Species × CaP × PHY			0.059	0.598	0.868	0.989	0.013	0.020	0.682

1 Calculated composition: CaP–, 4.1 g P/kg and 5.5 g Ca/kg; CaP+, 9.0 g P/kg and 12.0 g Ca/kg; PHY–, no supplemented phytase; PHY+: 1,500 FTU/kg supplemented phytase.

a-e Means within a column not sharing a common superscript differ (*P* < 0.05).

Prececal P digestibility was also significantly affected by a 3-way interaction (*P* = 0.020). In all PHY– treatments, prececal P digestibility was lower than in PHY+ treatments, except in turkeys fed CaP+, where phytase supplementation showed no effect. Prececal Ca digestibility was not significantly different between turkeys and broilers in CaP– treatments but was reduced by CaP+ to a greater extent in broilers than in turkeys (species × CaP: *P* < 0.001). Furthermore, phytase supplementation increased prececal Ca digestibility only when the CaP level was low (CaP × PHY: *P* < 0.001).

The InsP_6_ and Ins(1,2,4,5,6)P_5_ concentrations in crop content were significantly affected by the species  ×  PHY interaction (*P* < 0.001, Table 4), indicating that phytase addition caused a reduction in the concentration of these molecules in broilers but barely in turkeys. Ins(1,2,5,6)P_4_ was only detected in the PHY+ treatments, and its concentration was higher in broilers than in turkeys (*P* < 0.001). The MI concentration in the crop content was slightly but significantly higher in turkeys than in broilers (*P* = 0.001).

**Table 4:** Effect of Ca and P level (CaP) and phytase supplementation (PHY) on the concentrations of *myo*-inositol and inositol phosphates (µmol/g DM) in the crop of broilers and turkeys at 21 d of age.

Species	Treatment^1^		InsP_6_	Ins(1,2,4,5,6)P_5_	Ins(1,2,3,4,5)P_5_	Ins(1,2,3,4,6)P_5_	Ins(1,2,5,6)P_4_	*Myo*-inositol
Broiler	CaP–	PHY–	14.0	1.2	0.7^b^	0.3	< loq^2^	2.0
		PHY+	11.6	0.8	0.6bcd	< loq	2.4	2.2
	CaP+	PHY–	14.0	1.2	0.6^cd^	0.2	< loq	2.0
		PHY+	11.5	0.7	0.7^a^	< loq	2.6	2.0
Turkey	CaP–	PHY–	14.4	1.3	0.6^cd^	0.2	< loq	2.2
		PHY+	14.2	1.2	0.7^bc^	< loq	0.3	2.2
	CaP+	PHY–	14.4	1.2	0.6^d^	0.2	< loq	2.2
		PHY+	14.3	1.2	0.7^bc^	0.2	0.3	2.2

SEM			0.34	0.05	0.02	0.02	0.27	0.07

*P*-values								
Species			< 0.001	< 0.001	0.010	0.352	< 0.001	0.001
CaP			1.000	0.313	0.499	0.071	0.688	0.136
PHY			< 0.001	< 0.001	0.002	1.000		0.136
Species × CaP			0.807	0.313	0.181	0.352	0.829	0.136
Species × PHY			< 0.001	< 0.001	1.000			0.136
CaP × PHY			0.917	0.735	0.002			0.614
Species × CaP × PHY			0.834	0.313	0.010			0.614

1 Calculated composition: CaP–, 4.1 g P/kg and 5.5 g Ca/kg; CaP+, 9.0 g P/kg and 12.0 g Ca/kg; PHY–, no supplemented phytase; PHY+: 1,500 FTU/kg supplemented phytase.

2 loq = limit of quantification (0.3 µmol/g DM).

a-e Means within a column not sharing a common superscript differ (*P* < 0.05).

The InsP_6_ concentration in the gizzard content was lower in turkeys than in broilers in PHY– diets (5.7 µmol/g vs. 6.8 µmol/g) and reduced to a similar level (0.8 µmol/g and 0.6 µmol/g) in PHY+ diets (species × PHY: *P* < 0.001) (Table 5). The InsP_6_ concentration was also affected by CaP x PHY interaction (*P* = 0.003), and it was lower in CaP+PHY– (6.0 µmol/g) than in CaP–PHY– (6.6 µmol/g). In the treatments with supplemented phytase, the InsP_6_ concentration was higher in CaP+ (0.8 µmol/g) than CaP– (0.5 µmol/g). Ins(1,2,5,6)P_4_ was detected in the gizzard content only in the PHY+ treatment. The MI concentration in the gizzard content was affected by all two-way interactions. It was increased by the addition of phytase to a greater extent in broilers than in turkeys (*P* = 0.006 for the interaction) in CaP– but not CaP+ (*P* < 0.001 for the interaction).

**Table 5:** Effect of Ca and P level (CaP) and phytase supplementation (PHY) on the concentrations of *myo*-inositol and inositol phosphates (µmol/g DM) in the gizzard of broilers and turkeys at 21 d of age.

Species	Treatment^1^		InsP_6_	Ins(1,2,4,5,6)P_5_	Ins(1,2,3,4,5)P_5_	Ins(1,2,5,6)P_4_	InsP_3x_^2^	*Myo*-inositol
Broiler	CaP–	PHY–	7.0	0.4	0.3	n.d.^3^	n.d.	1.7
		PHY+	0.5	n.d.	n.d.	2.3	< loq^4^	2.7
	CaP+	PHY–	6.6	0.5	0.2	n.d.	n.d.	1.1
		PHY+	0.7	n.d.	0.1	3.9	< loq	1.2
Turkey	CaP–	PHY–	6.1	0.4	0.2	n.d.	n.d.	0.6
		PHY+	0.6	n.d.	n.d.	3.3	< loq	1.1
	CaP+	PHY–	5.3	0.4	0.2	n.d.	n.d.	0.6
		PHY+	1.0	n.d.	0.3	3.6	n.d.	0.6

SEM			0.21	0.03	0.03	0.24		0.07

*P*-values								
Species			0.004	0.018	1.000	0.172		< 0.001
CaP			0.374	0.771	0.367	0.001		< 0.001
PHY			< 0.001		0.546			< 0.001
Species × CaP			0.751	0.771	0.546	0.012		< 0.001
Species × PHY			< 0.001		0.001			0.006
CaP × PHY			0.003					< 0.001
Species × CaP × PHY			0.344					0.060

1 Calculated composition: CaP–, 4.1 g P/kg and 5.5 g Ca/kg; CaP+, 9.0 g P/kg and 12.0 g Ca/kg; PHY–, no supplemented phytase; PHY+: 1,500 FTU/kg supplemented phytase.

2 Ins(1,2,6)P_3_, Ins(1,4,5)P_3_, and Ins(2,4,5)P_3_ could not be differentiated due to co-elution and are thus referred to as InsP_3x_.

3 n.d. = not detectable (< 0.1 µmol/g DM).

4 loq = limit of quantification (0.3 µmol/g DM).

In the ileum, Ins(1,2,4,5,6)P_5_ concentrations were lowest in CaP– and unaffected by phytase or species, whereas in CaP+, concentrations where reduced by phytase in broilers but not in turkeys (*P* < 0.001, Table 6). Ins(1,2,3,4,5)P_5_ concentrations in the ileum were significantly affected by a CaP × PHY interaction (*P* < 0.001, Table 6) and were highest in CaP+PHY+ treatments, followed by CaP+PHY– and CaP–PHY+. CaP–PHY– was not different from CaP–PHY+ but lower than that of CaP+PHY–. The concentration of Ins(1,2,5,6)P_4_ in the ileum was the highest in broilers fed CaP+PHY+, and the PHY effect was lower in turkeys than in broilers (*P* < 0.001). InsP_3x_ in the ileum content was only detected in CaP+PHY+ treatments, and the concentration was higher in broilers than in turkeys (*P* < 0.001). The MI concentration in the ileum content was affected by a three-way interaction (*P* < 0.001). Broilers in the PHY+ treatments had higher MI concentrations than those in the PHY– treatments, with the highest concentration in CaP–. The MI concentration in the ileum of turkeys was lower than that in broilers. The MI concentration in the blood was significantly affected by the 3-way interaction (*P* < 0.001). The concentration was the lowest in CaP+PHY–. It was higher when CaP was low, PHY was added, or both. At CaP–PHY+, the highest plasma MI concentration was measured in both species, with the highest value in turkeys.

**Table 6:** Effect of Ca and P level (CaP) and phytase supplementation (PHY) on the concentrations of TiO_2_ (mg/g), *myo*-inositol, and inositol phosphates in the ileum (µmol/mg TiO_2_) and *myo*-inositol in blood plasma (µmol/ml) of broilers and turkeys at 21 d of age.

											*Myo*-inositol	
Species	Treatment^1^		TiO_2_	InsP_6_	Ins(1,2,4,5,6)P_5_	Ins(1,2,3,4,5)P_5_	Ins(1,2,3,4,6)P_5_	Ins(1,2,5,6)P_4_	Ins(1,2,3,4)P_4_	InsP_3x_^2^	Ileum	Blood
Broiler	CaP–	PHY–	15.3^b^	1.67^c^	0.03^de^	0.08	0.03	n.d.^3^	0.02	n.d.	0.67^b^	0.27^c^
		PHY+	15.8^b^	0.52^e^	0.03^e^	0.08	n.d.	0.05	0.01	n.d.	1.60^a^	0.33^b^
	CaP+	PHY–	16.1^b^	2.45^a^	0.18^a^	0.12	0.05	0.04	0.03	n.d.	0.14^d^	0.18^e^
		PHY+	17.1^a^	0.89^d^	0.11^c^	0.29	< loq^4^	0.66	0.07	0.37	0.31^c^	0.26^c^
Turkey	CaP–	PHY–	14.1^c^	1.76^c^	0.05^d^	0.06	0.03	n.d.	< loq	n.d.	0.11^d^	0.29^c^
		PHY+	13.2^cd^	0.99^d^	0.04^de^	0.11	< loq	0.04	0.01	n.d.	0.28^c^	0.43^a^
	CaP+	PHY–	12.4^d^	2.21^b^	0.15^b^	0.10	0.05	0.02	0.01	n.d.	0.12^d^	0.17^e^
		PHY+	14.0^c^	1.60^c^	0.14^b^	0.29	0.02	0.24	0.04	0.09	0.15^d^	0.21^d^

SEM			0.36	0.084	0.007	0.014	0.002	0.024	0.004	0.024	0.028	0.011

*P*-values												
Species			<0.001	<0.001	0.152	1.000	0.092	<0.001	0.004	<0.001	<0.001	0.055
CaP			0.181	<0.001	<0.001	<0.001	<0.001	<0.001	<0.001		<0.001	<0.001
PHY			0.015	<0.001	<0.001	<0.001	<0.001	<0.001	<0.001		<0.001	<0.001
Species × CaP			0.003	0.688	0.152	0.475	0.302	<0.001	0.004		<0.001	<0.001
Species × PHY			0.424	<0.001	0.001	0.095		<0.001	0.702		<0.001	0.260
CaP × PHY			0.003	0.232	0.002	<0.001			<0.001		<0.001	0.014
Species × CaP × PHY			0.030	0.012	<0.001	0.422					<0.001	<0.001

1 Calculated composition: CaP–, 4.1 g P/kg and 5.5 g Ca/kg; CaP+, 9.0 g P/kg and 12.0 g Ca/kg; PHY–, no supplemented phytase; PHY+: 1,500 FTU/kg supplemented phytase.

2 Ins(1,2,6)P_3_, Ins(1,4,5)P_3_, and Ins(2,4,5)P_3_ could not be differentiated due to co-elution and are thus referred to as InsP_3x_.

3 n.d. = not detectable (<0.1 µmol/g DM corresponds to <0.006 µmol/mg TiO_2_).

4 loq = limit of quantification (<0.3 µmol/g DM corresponds to 0.018 µmol/mg TiO_2_).

a-e Means within a column not sharing a common superscript differ (*P* < 0.05).

Performance traits were significantly affected by 3-way interactions (Table 7). Throughout all treatments, ADG (*P* < 0.001) and ADFI (*P* < 0.036) of broilers were higher than those of the turkeys. ADG and ADFI were lower in the CaP–PHY– treatments in each species than in the other treatments. The G:F ratio was generally higher in broilers than in turkeys. In broilers, G:F was similar in all treatments except for CaP–PHY–, which was lower. The G:F ratio in turkeys was lower in both CaP– treatments than in both CaP+ treatments (*P* < 0.004).

**Table 7:** Effect of Ca and P level (CaP) and phytase supplementation (PHY) on average daily gain (ADG), average daily feed intake (ADFI), gain to feed ratio (G:F) from 14-21 d of age, and length of small intestine and BW of broilers and turkeys (one bird per pen) at 21 d of age.

Species	Treatment^1^		ADG^2^ (g/d)	ADFI (g/d)	G:F (g/g)	Small intestine length (cm)	BW of the bird (kg)	Small intestine length (cm/kg BW)
Broiler	CaP–	PHY–	66.0^b^	77.0^b^	0.86^b^	141	852^b^	166
		PHY+	76.8^a^	84.7^a^	0.91^a^	142	1014^a^	140
	CaP+	PHY–	77.1^a^	85.5^a^	0.90^a^	150	971^a^	155
		PHY+	76.6^a^	84.6^a^	0.91^a^	150	1004^a^	150
Turkey	CaP–	PHY–	34.9^d^	48.0^d^	0.73^d^	108	533^d^	204
		PHY+	38.8^c^	52.5^c^	0.74^d^	111	556^d^	200
	CaP+	PHY–	39.9^c^	52.6^c^	0.76^c^	111	578^cd^	193
		PHY+	40.3^c^	52.5^c^	0.77^c^	113	637^c^	178

SEM			0.76	0.75	0.006	3.4	26.7	7.2

*P*-values								
Species			<0.001	<0.001	<0.001	<0.001	<0.001	<0.001
CaP			<0.001	<0.001	<0.001	0.023	0.004	0.110
PHY			<0.001	<0.001	<0.001	0.598	<0.001	0.017
Species × CaP			0.014	0.040	0.199	0.247	0.818	0.118
Species × PHY			<0.001	0.200	0.026	0.749	0.148	0.519
CaP × PHY			<0.001	<0.001	0.002	0.872	0.227	0.583
Species × CaP × PHY			<0.001	0.036	0.004	0.910	0.036	0.117

1 Calculated composition: CaP–, 4.1 g P/kg and 5.5 g Ca/kg; CaP+, 9.0 g P/kg and 12.0 g Ca/kg; PHY–, no supplemented phytase; PHY+: 1,500 FTU/kg supplemented phytase.

2 Average initial body weights per bird on d 14: 465 g (broilers) 295 g (turkeys).

a-d Means within a column not sharing a common superscript differ (*P* < 0.05).

The small intestine in total was longer in broilers than in turkeys (*P* < 0.001, Table 7). It was longer in CaP+ than CaP– treatments (*P* = 0.023), irrespective of species. When expressed per kilogram of BW, the small intestine was longer in turkeys than broilers (*P* < 0.001), not affected by CaP level, but shorter in PHY+ than PHY– treatments (*P* = 0.017).

The prececal digestibility of all analyzed AA was significantly affected by 3-way interactions (*P* ≤ 0.035, Tables 8 and 9). In broilers, prececal AA digestibility of most AA was higher in CaP–PHY+ (Ile, Leu, Lys, Met, Phe, Val, Tyr, Ala, and Ser) or CaP+PHY+ (His, Met, Thr, Cys, Ala, and Pro) than in CaP–PHY–. Prececal digestibility of Glx and Gly was not significantly affected in broilers. In turkeys, treatments with added phytase or CaP had lower prececal digestibility values for Arg, Ile, Leu, Met, Asp, Glu, Gly, and Pro compared to the CaP–PHY– treatment. However, the turkey CaP+Phy+ treatment had higher prececal digestibility values for Arg, His, Leu, Lys, Met, Thr, Val, Cys, Tyr, Ala, Asx, Gly, Pro, and Ser compared to the turkey CaP+PHY– treatment, reaching the same level as the turkey CaP–PHY– treatment. The treatment turkey CaP+PHY– showed the lowest prececal digestibility values of all analyzed AA.

**Table 8:** Effect of Ca and P level (CaP) and phytase supplementation (PHY) on prececal essential amino acid digestibility (%) of broilers and turkeys at 21 d of age.

Treatment^1^		Arg	His	Ile	Leu	Lys	Met^2^	Phe	Thr	Val
Broiler										
CaP–	PHY–	85.5^ab^	76.6^bc^	79.6^b^	79.3^bc^	84.8^bc^	90.8^bc^	80.1^bc^	71.6^bc^	77.9^bc^
	PHY+	86.9^a^	79.0^ab^	82.6^a^	82.4^a^	86.7^a^	92.2^a^	83.1^a^	74.2^ab^	80.9^a^
CaP+	PHY–	84.7^bc^	78.0abc	79.5^b^	79.9abc	84.6^bc^	91.5^ab^	80.3^bc^	72.3abc	78.3^bc^
	PHY+	85.9^ab^	79.7^a^	81.3^ab^	81.7^ab^	85.8^ab^	92.5^a^	82.0^ab^	74.6^a^	80.1^ab^
Turkey										
CaP–	PHY–	84.3^bc^	76.3^cd^	79.4^b^	79.0^c^	85.1abc	91.3^ab^	79.5bcd	71.9abc	77.5^cd^
	PHY+	82.1^de^	74.0^de^	76.9^c^	76.2^de^	83.8^cd^	89.9^cd^	77.0^de^	69.8^cd^	75.1^de^
CaP+	PHY–	80.9^e^	72.5^e^	75.9^c^	75.0^e^	82.9^d^	89.1^d^	75.6^e^	67.6^d^	73.8^e^
	PHY+	83.2^cd^	76.2^cd^	78.6^bc^	78.1^cd^	84.8^bc^	91.1abc	78.3cde	71.7abc	77.1^cd^

SEM		0.79	0.97	1.02	0.98	0.65	0.69	0.97	1.05	1.03

*P*-values										
Species		<0.001	<0.001	<0.001	<0.001	0.003	<0.001	<0.001	<0.001	<0.001
CaP		0.039	0.806	0.230	0.415	0.163	0.944	0.178	0.637	0.454
PHY		0.140	0.028	0.063	0.056	0.031	0.029	0.064	0.017	0.036
Species × CaP		0.765	0.145	0.851	0.467	0.955	0.145	0.498	0.208	0.621
Species × PHY		0.216	0.295	0.086	0.102	0.174	0.213	0.087	0.305	0.137
CaP × PHY		0.028	0.033	0.111	0.088	0.150	0.038	0.146	0.043	0.091
Species × CaP × PHY		0.017	0.009	0.016	0.012	0.029	0.007	0.015	0.026	0.012

1 Calculated composition: CaP–, 4.1 g P/kg and 5.5 g Ca/kg; CaP+, 9.0 g P/kg and 12.0 g Ca/kg; PHY–, no supplemented phytase; PHY+: 1,500 FTU/kg supplemented phytase.

2 Methionine determined as methionine sulfone.

a-e Means within a column not sharing a common superscript differ (*P* < 0.05).

**Table 9:** Effect of Ca and P level (CaP) and phytase supplementation (PHY) on prececal non-essential amino acid digestibility (%) of broilers and turkeys at 21 d of age.

Treatment^1^		Cys^2^	Tyr	Ala	Asx^3^	Glx^3^	Gly	Pro	Ser
Broiler									
CaP–	PHY–	64.2^b^	78.8^bc^	76.3^b^	73.8abc	82.5abc	71.4^a^	77.2^bc^	74.1^bc^
	PHY+	68.1^ab^	81.6^a^	79.5^a^	76.0^a^	84.4^a^	73.9^a^	79.1^ab^	77.0^a^
CaP+	PHY–	68.4^ab^	79.1^bc^	77.0^ab^	73.1^bc^	82.0abc	72.1^a^	77.6abc	74.4abc
	Phy+	70.5^a^	81.0^ab^	79.1^a^	75.2^ab^	83.6^ab^	74.0^a^	79.8^a^	76.6^ab^
Turkey									
CaP–	PHY–	54.1^cd^	79.1^bc^	77.2^ab^	74.0^ab^	81.3^bc^	71.4^a^	75.5^cd^	73.6^cd^
	Phy+	50.0^de^	76.8^cd^	74.4^bc^	71.4^cd^	78.1^d^	68.7^bc^	72.6^ef^	71.1^de^
CaP+	PHY–	46.8^e^	74.9^d^	73.1^c^	70.1^d^	77.2^d^	66.7^c^	70.5^f^	68.5^e^
	Phy+	55.2^c^	77.7^c^	76.9^ab^	73.0^bc^	79.8^cd^	71.1^ab^	74.1^de^	72.7^cd^

SEM		1.72	0.94	1.07	1.01	0.99	1.12	0.99	1.05

*P*-values									
Species		<0.001	<0.001	<0.001	<0.001	<0.001	<0.001	<0.001	<0.001
CaP		0.326	0.164	0.631	0.155	0.144	0.550	0.360	0.194
PHY		0.027	0.042	0.030	0.074	0.248	0.037	0.073	0.017
Species × CaP		0.062	0.211	0.506	0.718	0.682	0.262	0.082	0.214
Species × PHY		0.717	0.096	0.144	0.128	0.110	0.328	0.197	0.210
CaP × PHY		0.023	0.089	0.063	0.043	0.038	0.026	0.014	0.035
Species × CaP × PHY		0.003	0.019	0.011	0.035	0.023	0.008	0.021	0.010

1 Calculated composition: CaP–, 4.1 g P/kg and 5.5 g Ca/kg; CaP+, 9.0 g P/kg and 12.0 g Ca/kg; PHY–, no supplemented phytase; PHY+: 1,500 FTU/kg supplemented phytase.

2 Cysteine determined as cysteine sulfone.

3 The amide residue in the side group of asparagine and glutamine is lost during acid hydrolysis and aspartic acid and glutamic acid are formed. Hence, aspartic acid together with asparagine (Asx) and glutamic acid together with glutamine (Glx) was analyzed.

a-f Means within a column not sharing a common superscript differ (*P* < 0.05).

## DISCUSSION

The hypothesis that broilers exert an overall higher capability of InsP_6_ degradation than turkeys when fed the same feed must be rejected for 3-wk-old birds as there were no differences in jejunal mucosal phosphatase activity, prececal InsP_6_ disappearance, InsP_3-6_ concentrations in the digesta along the digestive tract, and prececal P and Ca digestibility between broilers and turkeys fed the CaP–PHY– diet. Broilers showed higher prececal InsP_6_ disappearance than turkeys only when phytase was supplemented. The similarities between broilers and turkeys found in the CaP–PHY– treatments indicate that 3-wk-old broilers and turkeys had the same endogenous InsP_6_ degradation when fed diets with low P and Ca concentrations and no phytase supplementation. These findings are inconsistent with previous comparative studies that reported higher prececal InsP_6_ disappearance in broilers than in turkeys (Ingelmann et al., 2019; Olukosi et al., 2020). However, in these studies, species-specific diets were compared, which differed markedly in the concentrations of nutrients such as P and Ca. In chickens, adverse effects on phytate degradation have been reported for high dietary concentrations of mineral P (Olukosi et al., 2013; Shastak et al., 2014; Walk et al., 2014; Zeller et al., 2015b; Sommerfeld et al., 2018; Siegert et al., 2019) and Ca (Tamim et al., 2004; Sommerfeld et al., 2018). Possible reasons for these adverse effects are the end-product inhibition of phytase by mineral P (Greiner et al., 1993; Zeller et al., 2015b) and precipitation and subsequent inaccessibility for the enzyme caused by chelating Ca^2+^ ions (Walk et al., 2012; Sommerfeld et al., 2018). There is no indication that these mechanisms do not affect turkeys. In the present study, high CaP levels markedly reduced prececal InsP_6_ disappearance in broilers and turkeys compared to low CaP levels, although slightly more in broilers. Hence, it is likely that in the previously mentioned comparative studies, differences in P and Ca concentrations of the feed were largely responsible for the observed differences in prececal InsP_6_ disappearance and not the inherent capabilities of the animals.

In broilers, Rodehutscord et al. (2012) found a slight reduction of prececal P digestibility when dietary P concentrations increased incrementally to 8.1 g/kg using MCP. Further, when dietary P concentrations increased incrementally to 7.7 g/kg DM, Rodehutscord and Dieckmann (2005) reported a reduction in the percentage of retained P in broilers and an increase in the percentage of retained P in turkeys, using the same feed for both species. Thus, the high levels of supplemented P and Ca in the present trial were likely absorbed more by turkeys than broilers, as in PHY– treatments, increased dietary Ca and P concentrations led to higher prececal P digestibility in turkeys but not in broilers. In addition, Ca digestibility in the CaP+ treatments was higher in turkeys than in broilers. This led to higher Ca and P concentrations in the ileum of broilers than in turkeys in the CaP+ treatments, whereas in CaP– treatments Ca and P concentrations in the ileum were the same for both species (data not shown). Thus, the greater effect of CaP+ on prececal InsP_6_ disappearance in broilers than in turkeys is assumed to be caused by the aforementioned adverse effects of higher P and Ca concentrations in the gut of broilers than in turkeys. This is assumed to have led to the significantly lower prececal InsP_6_ disappearance in broilers than in turkeys in the CaP+PHY– treatment. A negative value for prececal InsP_6_ disappearance in broilers was calculated for the CaP+PHY– treatment. Because InsP_6_ formation in the digestive tract is unlikely to occur, this effect was likely an artefact of the marker method. Therefore, it was assumed that, in this treatment, there was very little or no prececal InsP_6_ disappearance.

Sommerfeld et al. (2019) showed that up to 42% of prececal InsP_6_ disappearance can be attributed to endogenous mucosal phosphatase activity in broilers. In the present study, similar jejunal mucosal phosphatase activities between turkeys and broilers in the CaP–PHY– treatment coincided with similar prececal InsP_6_ disappearance and prececal P digestibility; however, it remains unclear to what extent the mucosal phytase activity contributed to phytate degradation, because enzymes from resident microbiota or the feed ingredients, not analyzed by the product-specific ELISA method, might have been involved. The higher mucosal phosphatase activity caused by the supplemented phytase could have been triggered by the relatively high concentrations of Ins(1,2,5,6)P_4_ found in the gizzard, which Walk et al. (2018) hypothesized is due to a triggering mechanism of lower InsP. Similar to the treatments without phytase supplementation, the contribution of mucosal phosphatase to phytate degradation is unclear.

As expected, supplementation with phytase increased prececal InsP_6_ degradation in both species. However, the effect of phytase supplementation was much greater in broilers than in turkeys. The InsP_6_ concentration in the crop was reduced by phytase supplementation only in broilers. When the crop was opened, we observed that feed pellets were still visible in turkeys other than broilers, whose crop content was a moist mash. Also, there was a difference in weight loss of digesta samples from the crop during freeze-drying between broilers and turkeys (broilers: 65.7%, turkeys: 36.8% weight loss, *P* < 0.001, data not shown). Thus, the difference in InsP_6_ disappearance might have been caused by the higher water content in the crop of broilers compared to turkeys, as supplemented phytase might have lacked a medium to access phytate in turkeys. The lack of an effect of phytase supplementation on InsP_6_ concentration in the crop of turkeys may explain the lower prececal InsP_6_ disappearance in turkeys compared to broilers. However, differences in prececal InsP_6_ disappearance were not as severe as the differences in crop InsP_6_ concentration might suggest. Possible explanations for this could be the following: Lower pH in the gizzard and duodenum of turkeys compared to broilers could have led to higher InsP_6_ solubility (Grynspan and Cheryan, 1983) and, consequently, more InsP_6_ degradation in these sections. In addition, according to Menezes-Blackburn et al. (2015), pH was slightly closer to the optimum of the supplemented phytase in the gizzard and duodenum of turkeys than in broilers. Furthermore, differences in retention time could not be ruled out as a reason for the species effect.

In the crop of broilers, there was a reduction in InsP_6_ and Ins(1,2,4,5,6)P_5_ concentrations when supplemented with phytase. The small variation in Ins(1,2,3,4,5)P_5_ concentration in the crop of all treatments indicates that the release of the first two phosphate groups from InsP_6_ occurred at a similar pace. Elevated Ins(1,2,5,6)P_4_ concentrations in the digestive tract in PHY+ treatments indicate that the supplemented phytase was limited in activity in the third degradation step. This is consistent with results from previous studies using 6-phytases in broilers (Walk et al., 2014; Zeller et al., 2015a,b; Krieg et al., 2020; Olukosi et al., 2020; Kristoffersen et al., 2021) and turkeys (Olukosi et al., 2020). However, in the terminal ileum Ins(1,2,5,6)P_4_ concentrations were substantially elevated only in CaP+PHY+ treatments, indicating inhibitory effects of high CaP level on mucosal phosphatase activity.

**Figure 1:**
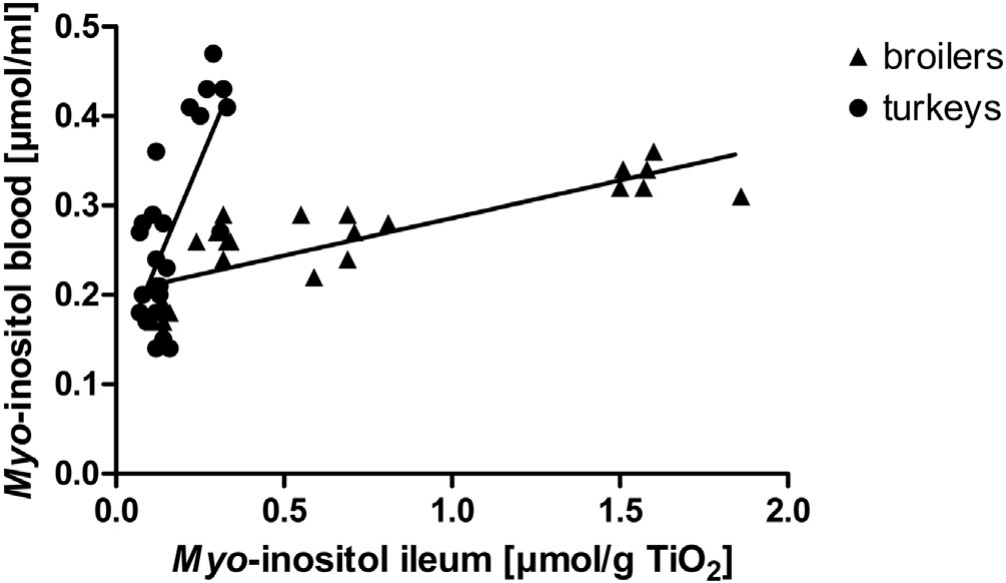
*Myo*-inositol concentrations in ileum digesta (per g TiO_2_) and blood of broilers and turkeys fed the experimental diets at 21 d of age. Linear regression broilers: y = 0.08 x + 0.20, r^2^ = 0.69. Linear regression turkeys: y = 0.89 x + 0.13, r^2^ = 0.53.

The higher MI concentrations in the ileum of broilers compared to turkeys appear to be inconsistent with similarly high prececal InsP_6_ disappearance and mucosal phosphatase activity, especially in the CaP–PHY– treatment. However, in the blood, MI concentrations were similar in both species, except for the treatment CaP–Phy+, where turkeys exerted a higher MI concentration than broilers, even though their prececal InsP_6_ disappearance was lower. In addition, a linear relationship between MI concentrations in the blood and ileum was observed in both species (Figure 1); however, the regression slope was greater in turkeys than in broilers. Thus, it is speculated that the lower concentration of MI in the ileum of turkeys was caused by a higher uptake rate, a different uptake location in the small intestine, a different turnover rate in the enterocytes, or a different transport rate from the blood to organs of turkeys. A concentration-dependent increase in active MI transport in the small intestine of broilers has been previously discussed (Walk et al., 2018), but the authors are unaware of comparable information on turkeys.

P digestibility increased with phytase supplementation following an increase in InsP_6_ degradation. In addition, Ca digestibility increased with phytase supplementation, possibly due to fewer Ca^2+^ ions forming complexes with phytate and a higher metabolic Ca demand when more P is absorbed and retained in bones.

As reviewed by Rodehutscord et al. (2022), phytase supplementation increased AA digestibility in some, but not all, studies. In the present study, AA digestibility in broilers was significantly increased in 9 out of 17 analyzed AA, but only when the CaP level was low. This indicates that AA digestibility in broilers was affected by prececal InsP_6_ disappearance and unknown factors linked to dietary P and Ca concentrations. In turkeys, the CaP level and supplemented phytase effects were considerably different, as an increase in prececal AA digestibility due to phytase supplementation was only seen when the CaP level was high (14 out of 17 analyzed AA). In contrast, when the CaP level was low, phytase supplementation decreased prececal AA digestibility in 8 out of 17 analyzed AA. This might have been related to different digesta passage rate owing to differences in small intestine lengths between PHY+ and PHY– (Table 7). In CaP+ treatments such effect might have been compensated by higher absolute length of the small intestine. In any case, differences indicate that prececal AA digestibility is affected differently by P, Ca, and InsP_6_ concentrations in turkeys and broilers.

As 2 species were compared that differ markedly in their maturation rates (Zuidhof et al., 2014; Tůmová et al., 2020), it is necessary to evaluate whether observed differences in phytate degradation and nutrient digestibility can be attributed to species effects or if effects of different physiological development were involved. Thus, a second trial was conducted with 6 wk-old turkeys and broilers, receiving similar dietary treatments as in the present trial (Novotny et al., 2023). Many findings of the present study were confirmed in 6 wk-old birds. However, it was found that age affected turkeys and broilers differently regarding endogenous mucosal phosphatase activity and prececal InsP_6_ disappearance, both increasing with age in turkeys. This resulted in higher prececal InsP_6_ disappearance in 6 wk-old turkeys than 6 wk-old broilers, when no phytase was supplemented. As such, higher mucosal phosphatase activity coincided with higher prececal InsP_6_ disappearance. Comparisons are being discussed in more detail in the companion paper (Novotny et al., 2023).

In conclusion, there was no difference in the endogenous InsP_6_ degradation capabilities between 3-wk-old broilers and turkeys. Previously reported differences between species were possibly owing to differences in diet composition. Furthermore, the effect of phytase supplementation on prececal InsP_6_ disappearance was greater in broilers than in turkeys, which might be linked to better crop conditions. Also, the uptake and metabolism of MI, as well as the effects of phytase supplementation on AA digestibility, deserve further research, as apparent species differences in these traits require elucidation. Further research is also necessary to clarify the relevance of age of the birds for the studied traits.
